# An analysis of vascular properties using pulse wave analysis in patients with vasovagal syncope

**DOI:** 10.1002/clc.23380

**Published:** 2020-06-18

**Authors:** Ji‐Hun Jang, Jin‐Hee Park, Kyu‐Yong Ko, Yong‐Soo Baek, Sung‐Woo Kwon, Sang‐Don Park, Sung‐Hee Shin, Seong‐Ill Woo, Jun Kwan, Dae‐Hyeok Kim

**Affiliations:** ^1^ Department of Cardiology Inha University Hospital Cardiovascular Center Incheon Republic of Korea

**Keywords:** augmentation index, head‐up tilt, pulse wave analysis, pulse wave velocity, vasovagal syncope

## Abstract

**Background:**

Vasovagal syncope (VVS) is a common cause of recurrent syncope. Nevertheless, the exact hemodynamic mechanism has not been elucidated. Pulse wave analysis (PWA) is widely used to evaluate vascular properties, as it reflects the condition of the entire arterial system.

**Hypothesis:**

Cardiovascular autonomic modulation may influence the hemodynamic mechanism and result in different vascular properties between VVS patients and healthy individuals.

**Methods:**

We enrolled consecutive patients diagnosed with VVS on head‐up tilt testing from January 2014 to August 2019. Healthy subjects were enrolled as the control group. We performed PWA on all participants. Using propensity score matching, we assembled a study population with similar baseline characteristics and compared hemodynamic parameters.

**Results:**

A total of 111 VVS patients (43 ± 18 years, 72 females) and 475 healthy control subjects (48 ± 13 years, 192 females) were enrolled. Compared to the healthy control subjects, the VVS patients had a higher augmentation index (AIx) adjusted to a heart rate of 75 beats per minute (AIx@HR75, 20.5 ± 13.1% vs 16.7 ± 11.9%, *P* = .003). After 1:1 matched comparison (111 matched control), VVS patients consistently showed higher AIx@HR75 (20.5 ± 13.1% vs 16.7 ± 12.9%, *P* = .02) than the matched control group. According to age distribution, VVS patients showed significantly higher AIx@HR75 (10.6 ± 11.7% vs 2.5 ± 11.1%, *P* = .01) in a young age (15‐33 years) group.

**Conclusions:**

VVS patients had greater arterial stiffness than healthy subjects. This is one of the plausible mechanisms of the pathophysiology of VVS.

## 1 INTRODUCTION

Syncope is defined as transient loss of consciousness due to cerebral hypoperfusion, characterized by rapid onset, short duration, and complete spontaneous recovery.^1^, ^2^ According to recent guidelines, syncope can be divided into three main groups: reflex, cardiovascular, and secondary to orthostatic hypotension. Vasovagal syncope (VVS), mediated by the vasovagal reflex, is the most common presentation of syncope in the general population.^1^, ^3^ Recently, the consensus precise pathophysiological mechanisms underlying VVS suggested that the autonomic nervous system is the final common pathway leading to syncope. Therefore, cardiovascular autonomic modulation may play a role in the occurrence of syncope.^3^, ^4^, ^5^ Nevertheless, the exact hemodynamic mechanisms and the relationship with autonomic regulation has not been elucidated.^6^, ^7^ Arterial pulse wave analysis (PWA) is a noninvasive index of arterial distensibility now generally advocated for the assessment of cardiovascular risk as well as for measuring blood pressure. It reflects central and peripheral vascular properties by measuring arterial stiffness and elastic compliance.^8^, ^9^, ^10^ Therefore, we investigated central hemodynamics using PWA and compared VVS patients with healthy control subjects. We hypothesized that cardiovascular autonomic modulation may influence the hemodynamic mechanism and that vascular properties differed between VVS patients and healthy individuals.

## 2 METHODS

### 2.1 Study design

We enrolled 133 consecutive patients diagnosed with VVS at our institution from January 2014 to August 2019. The diagnosis of VVS was made using the head‐up tilt (HUT) test based on the current diagnostic guidelines.^1^, ^2^ A positive response is defined as inducible presyncope or syncope associated with hypotension, with or without bradycardia (less commonly asystole). All patients were free from medication that could influence vascular properties and autonomic nervous system, including antihypertensive and neuromuscular drugs. We excluded patients with conditions that can affect vascular properties and hemodynamics (eg, hypertension, diabetes mellitus, renal disease, cerebrovascular disease, coronary or peripheral vascular disease, and structural heart disease) and those with arrhythmias and psychiatric disorders. We defined and classified VVS based on the modified Vasovagal Syncope International Study criteria as follows: type I, mixed; type II, cardio‐inhibitory; type III, vasodepressor.^11^

As the control group, we enrolled healthy subjects who were free from any syncope or presyncope, or who showed negative HUT test. Additionally, subjects without VVS (eg, postural orthostatic tachycardia syndrome and orthostatic intolerance without tachycardia) on the HUT test were included in the control group.

The study design was approved by the Institutional Review Board of Inha University Hospital, Incheon, South Korea and was conducted in compliance with the ethical principles outlined in the Declaration of Helsinki (INHAUH 2019‐08‐012).

### 2.2 HUT test

The tilt table test was performed with the patient in fasting state for 2 to 4 hours before the test was conducted in a quiet, closed room according to the recent standardized protocol. The patient was stabilized in the supine position for 5 minutes without venous cannulation and for 20 minutes with venous cannulation. The tilted angle was maintained between 60° and 70° for 20 minutes to induce syncope. If syncope was not induced, we started isoproterenol challenge at an incremental infusion rate from 1 to 3 μg/minute to increase the average heart rate by about 20% to 25% over baseline. Twelve‐lead electrocardiogram (ECG) tracings and BP were measured every 2.5 minutes. The test was continued until the development of positive signs or completion of the protocol.

### 2.3 Pulse wave analysis

To investigate vascular properties, we used the SphygmoCor (AtCor Medical Pty Ltd Head Office, West Ryde, Australia) system in all subjects. The examination was performed in a quiet room with a comfortable room temperature and the patient in a supine position.

The carotid and femoral pulse waves were analyzed, estimating the delay in theECG wave and calculating the pulse wave velocity (PWV). In addition to the estimation of radial and central blood pressure, central hemodynamic parameters including ejection duration (ED), the time to the peak/shoulder of the first (T1), and second pressure wave components (T2) during systole, the time to return of the reflected pressure (Tr) wave, P1 height, aortic pulse pressure (PP), augmentation pressure (AP), augmentation index (AIx), subendocardial viability ratio (SEVR) were estimated from the aortic wave morphology.^12^, ^13^ The P1 height is defined as the difference between the central pressure at T1 and the diastolic pressure. The AIx was defined as the augmented pressure (magnitude of wave reflection) divided by PP: AIx = pressure increase/PP × 100. Because AIx is influenced by heart rate, we estimated AIx adjusted to a heart rate of 75 beats per minute (AIx@HR75).

### 2.4 Statistical analyses

Data were expressed for continuous variables as mean ± SD and categorical variables as counts and percentages. The Student's *t* test and Pearson's chi‐square test were used to comparing each parameter as needed. The Mann‐Whitney *U* test was used for skewed variables, and Fisher's exact test was used when the expected frequency was lower than 5.

Considering that PWA is affected by physical characteristics, a propensity score matching strategy was used to minimize confounders for adjusted by age, gender, mean blood pressure, heart rate, height, and weight. Participants were matched using 1‐to‐1 nearest‐neighbor matching without replacement. A caliper width of 0.2 of the SD of the logit of the propensity score was used for the developed propensity score.

For all tests, a *P* value less than .05 was considered statistically significant. All statistical analyses were performed using R statistical software (version 3.4.1; R Foundation for Statistical Computing, Vienna, Austria).

## 3 RESULTS

### 3.1 Study population

We initially enrolled 133 patients with VVS. Among them, we excluded 22 patients according to the exclusion criteria. Finally, 111 VVS patients were included in our study. A total of 475 healthy control subjects were enrolled. Among them, 39 subjects underwent HUT test and showed negative results.

Prior to propensity score matching, significant differences in demographics and PWA parameters of patients were documented (Table 1). The control group was older than the VVS group (48.3 ± 13.3 vs 42.6 ± 17.6, *P* = .002) and was taller, heavier, and had higher baseline heart rates (166.5 ± 9.4 cm vs 163.7 ± 9.1 cm, *P* = .004; 68.0 ± 13.5 kg vs 60.7 ± 9.6 kg, *P* < .001; 69.2 ± 11.5 bpm vs 65.8 ± 10.6 bpm, *P* = .004, respectively) whereas the VVS group had more female patients (64.9% vs 40.4%, *P* < .001). Because we performed the matching process based on baseline characteristics including age, gender, height, weight, heart rate, and mean blood pressure, the procedure yielded 111 well‐matched pairs. After propensity score matching, both groups were well matched, with no significant differences in baseline characteristics (Table 1).

**TABLE 1:** Baseline characteristics and pulse wave analysis by VVS group or healthy control subjects in the overall study population and 1:1 matched study population

Parameters	Overall			Matched		
	VVS (n = 111)	Control (n = 475)	*P* value	VVS (n = 111)	Control (n = 111)	*P* value
Baseline characteristics						
Age (y)	43 ± 18	48 ± 13	.002	43 ± 18	43 ± 14	.90
Female, n (%)	72 (64.9)%	192 (40.4)%	<.001	72 (64.9)%	73 (65.8)%	.99
Height (cm)	163.7 ± 9.1	166.5 ± 9.4	.004	163.7 ± 9.1	163.8 ± 9.4	.90
Weight (kg)	60.7 ± 9.6	68.0 ± 13.5	<.001	60.7 ± 9.6	61.7 ± 12.3	.95
BSA (m^2^)	1.7 ± 0.2	1.8 ± 0.2	<.001	1.7 ± 0.2	1.7 ± 0.2	.87
BMI (kg/m^2^)	22.6 ± 2.7	24.4 ± 3.5	<.001	22.6 ± 2.7	22.9 ± 3.4	.77
Smoking (%)	10 (9.0)%	64 (13.5)%	.26	10 (9.0)%	11 (9.9%)	.99
Heart rate, bpm	65.8 ± 10.6	69.2 ± 11.5	.004	65.8 ± 10.6	65.8 ± 10.2	.74
Pulse wave analysis						
Radial BP (mm Hg)						
Systolic	118.3 ± 14.2	118.4 ± 13.2	.84	118.3 ± 14.2	116.7 ± 13.5	.56
Diastolic	72.3 ± 8.5	73.5 ± 9.0	.21	72.3 ± 8.5	72.7 ± 9.0	.71
MP	89.3 ± 10.3	89.6 ± 10.0	.77	89.3 ± 10.3	88.7 ± 10.5	.69
PP	46.0 ± 10.8	45.0 ± 9.8	.34	46.0 ± 10.8	43.9 ± 8.9	.15
Aortic BP (mm Hg)						
Systolic	108.8 ± 15.2	107.7 ± 12.9	.46	108.8 ± 15.2	106.9 ± 14.1	.63
Diastolic	73.4 ± 8.5	74.6 ± 9.1	.20	73.4 ± 8.5	73.7 ± 9.1	.78
MP	89.3 ± 10.3	89.4 ± 10.1	.90	89.3 ± 10.3	88.7 ± 10.4	.68
PP	35.5 ± 11.3	33.2 ± 9.1	.05	35.5 ± 11.3	33.5 ± 9.4	.26
T1 (m/s)	106.9 ± 12.0	110.7 ± 13.0	.005	106.9 ± 12.0	110.5 ± 14.3	.03
T2 (m/s)	229.8 ± 27.0	220.7 ± 25.7	.001	229.8 ± 27.0	226.2 ± 26.1	.21
Tr (m/s)	143.3 ± 15.1	145.3 ± 15.8	.22	143.3 ± 15.1	145.3 ± 17.2	.30
P1 height (mm Hg)	25.6 ± 5.9	25.5 ± 5.6	.86	25.6 ± 5.9	24.9 ± 5.3	.41
AP	9.8 ± 7.6	7.4 ± 6.0	.003	9.8 ± 7.6	8.0 ± 6.4	.11
Aortic AIx (%)	24.7 ± 14.2	20.5 ± 13.5	.003	24.7 ± 14.2	22.2 ± 14.0	.16
AIx@HR75 (%)	20.5 ± 13.1	16.7 ± 11.9	.003	20.5 ± 13.1	16.7 ± 12.9	.02
ED (m/s)	36.2 ± 4.6	36.9 ± 5.4	.18	36.2 ± 4.6	35.8 ± 4.1	.69
SEVR (%)	152.5 ± 27.8	150.7 ± 30.1	.56	152.5 ± 27.8	156.9 ± 27.7	.24
PWV (m/s)	6.6 ± 1.5	6.7 ± 1.3	.61	6.6 ± 1.5	6.2 ± 1.2	.07
Operator index	96.0 ± 5.0	96.1 ± 4.7	.84	96.0 ± 5.0	95.3 ± 5.1	.16

Abbreviations: AIx, augmentation index; AIx@HR75, augmentation index adjusted to a heart rate of 75 beats per minute; AP, augmentation pressure; BMI, body mass index; BP, blood pressure; bpm, beats per minute; BSA, body surface area; ED, ejection duration; MP, mean pressure; PP, pulse pressure; PWV, pulse wave velocity; SEVR, subendocardial viability ratio; T1, time at the first peak/shoulder during systole (outgoing pressure wave); T2, time at the second peak/shoulder during systole (reflected pressure wave); Tr, time to return of the reflected pressure; VVS, vasovagal syncope.

### 3.2 Hemodynamic parameters from PWA

Before matching, the radial and aortic blood pressure did not differ between the two groups. However, the VVS group showed shorter T1 (106.9 ± 12.0 m/s vs 110.7 ± 13.0 m/s, *P* = .005) and prolonged T2 (229.8 ± 27.0 m/s vs 220.7 ± 25.7 m/s, *P* = .001) than the control group, whereas Tr was comparable. P1 height, ED, and SEVR showed no significant differences. AIx and AIx@HR75 were significantly higher in the VVS group (24.7 ± 14.2% vs 20.5 ± 13.5%, *P* = .003; 20.5 ± 13.1% vs 16.7 ± 11.9%, *P* = .003, respectively). PWV showed no significant difference (*P* = .61).

**FIGURE 1:**
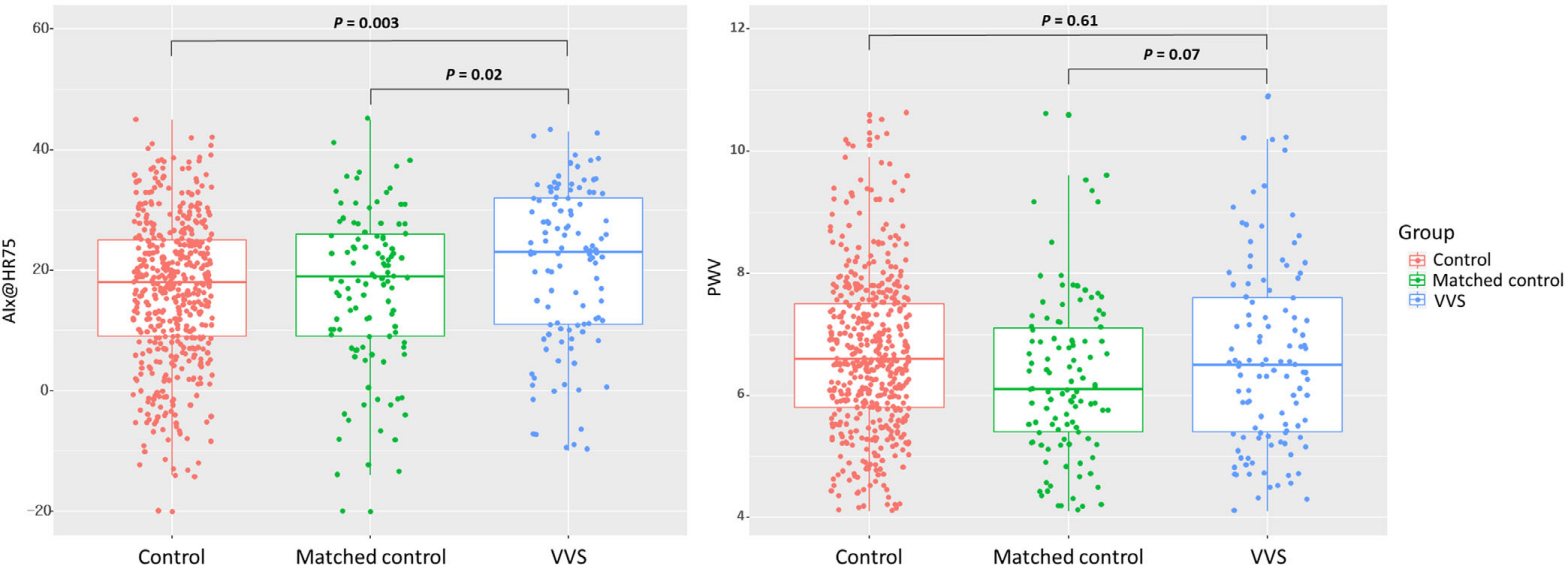
Comparison of augmentation index adjusted to a heart rate of 75 beats/minute (AIx@HR75) and pulse wave velocity (PWV) among each group

After a matched comparison, the VVS group still showed shorter T1 (106.9 ± 12.0 m/s vs 110.5 ± 14.3 m/s, *P* = .03), whereas T2 was not significantly different. AIx@HR75 was significantly greater in the VVS group (24.7 ± 14.2% vs 22.2 ± 14.0%, *P* = .02). Although there was no statistically significance, PWV was higher in the VVS group than in the matched control group (6.6 ± 1.5 m/s vs 6.2 ± 1.2 m/s, *P* = .07). Figure 1 shows a box plot for AIx@HR75 and PWV for each group.

We analyzed hemodynamic parameters according to VVS classification. Fourteen patients were mixed type, 12 were cardio‐inhibitory, and 85 were vasodepressor. Baseline characteristics were comparable among the groups. In terms of hemodynamic parameters, T1 value was insignificantly shortest in vasodepressor. The mixed type showed the highest AIx@HR75, and the cardio‐inhibitory type showed the highest PWV value. However, there was no statistical significance (Table S1).

### 3.3 PWA according to the age distribution

According to the age distribution, we divided into three groups in the study population and compared PWA parameters (Table 2): young age group (15‐33 years), middle‐age group (34‐54 years), and old age group (54‐79 years).

**TABLE 2:** Baseline characteristics and pulse wave analysis according to the age distribution in the 1:1 matched study population

Parameters	Young age (15‐33 years)			Middle age (34‐54 years)			Old age (54‐79 years)		
	VVS (n = 39)	Control (n = 28)	*P* value	VVS (n = 39)	Control (n = 59)	*P* value	VVS (n = 33)	Control (n = 24)	*P* value
Baseline characteristics									
Age (y)	23 ± 5	25 ± 5	.20	44 ± 6	44 ± 6	.73	64 ± 6	61 ± 5.	.08
Female, n (%)	27 (69.2%)	16 (57.1%)	.45	27 (69.2%)	36 (61.0%)	.54	18 (54.5%)	21 (87.5%)	.02
Height (cm)	166.5 ± 8.5	169.1 ± 10.0	.25	163.1 ± 9.0	164.5 ± 7.5	.41	161.0 ± 9.2	156.0 ± 7.9	.04
Weight (kg)	59.5 ± 8.8	61.6 ± 16.3	.54	61.4 ± 9.8	62.7 ± 11.7	.55	61.3 ± 10.3	59.2 ± 7.7	.40
BSA (m^2^)	1.7 ± 0.2	1.7 ± 0.2	.49	1.7 ± 0.2	1.7 ± 0.2	.51	1.7 ± 0.2	1.6 ± 0.1	.16
BMI (kg/m^2^)	21.4 ± 2.4	21.4 ± 4.2	.94	23.0 ± 2.8	23.0 ± 2.9	.98	23.5 ± 2.4	24.2 ± 2.8	.34
Smoking (%)	3 (7.7%)	5 (17.9%)	.26	3 (7.7%)	6 (10.2%)	.99	4 (12.1%)	0 (0.0%)	.13
Heart rate, bpm	69.9 ± 12.3	65.4 ± 9.0	.10	64.2 ± 8.9	67.0 ± 9.7	.14	62.7 ± 8.9	63.4 ± 12.5	.81
Pulse wave analysis									
Radial BP (mm Hg)	112.8 ± 11.1	110.0 ± 14.5	.38	115.8 ± 13.7	117.9 ± 12.8	.44	127.8 ± 13.6	121.5 ± 11.3	.07
Systolic	69.4 ± 7.5	66.2 ± 9.0	.12	72.3 ± 8.5	75.4 ± 8.6	.08	75.7 ± 8.4	73.8 ± 6.1	.34
Diastolic	84.0 ± 7.7	79.9 ± 9.2	.05	89.2 ± 10.6	91.3 ± 9.8	.32	95.6 ± 9.3	92.7 ± 7.3	.20
MP	112.8 ± 11.1	110.0 ± 14.5	.38	115.8 ± 13.7	117.9 ± 12.8	.44	127.8 ± 13.6	121.5 ± 11.3	.07
PP	43.4 ± 9.6	43.8 ± 10.9	.87	43.5 ± 7.6	42.5 ± 7.4	.52	51.9 ± 13.1	47.2 ± 9.1	.14
Aortic BP (mm Hg)									
Systolic	98.9 ± 8.1	95.1 ± 11.3	.12	108.3 ± 14.4	108.8 ± 12.8	.85	121.3 ± 13.9	115.9 ± 11.0	.12
Diastolic	70.5 ± 7.5	67.0 ± 8.9	.08	73.3 ± 8.6	76.5 ± 8.6	.08	76.8 ± 8.5	74.8 ± 6.2	.31
MP	84.0 ± 7.7	79.9 ± 9.2	.05	89.2 ± 10.6	91.3 ± 9.8	.31	95.6 ± 9.3	92.5 ± 7.1	.18
PP	28.3 ± 5.3	29.6 ± 9.5	.52	35.0 ± 8.7	32.3 ± 7.5	.11	44.5 ± 12.9	40.9 ± 9.8	.26
T1 (m/s)	106.5 ± 12.6	118.0 ± 21.7	.02	108.0 ± 12.9	108.2 ± 10.7	.91	105.9 ± 10.2	107.1 ± 6.5	.61
T2 (m/s)	210.2 ± 28.9	205.4 ± 27.2	.49	238.2 ± 17.8	229.1 ± 20.5	.03	243.0 ± 20.3	243.2 ± 22.2	.97
Tr (m/s)	149.1 ± 16.2	159.8 ± 23.7	.06	142.9 ± 16.0	142.6 ± 12.1	.91	137.6 ± 10.1	136.5 ± 8.9	.69
P1 height (mmHg)	24.6 ± 5.5	25.9 ± 6.6	.38	24.1 ± 4.1	24.0 ± 4.5	.90	28.7 ± 7.0	26.1 ± 5.4	.14
AP	3.6 ± 3.1	2.0 ± 3.5	.05	10.8 ± 6.6	8.1 ± 4.9	.03	15.9 ± 7.0	14.5 ± 5.7	.44
Aortic AIx (%)	12.8 ± 10.3	7.1 ± 10.4	.03	28.6 ± 13.2	24.1 ± 11.1	.07	34.3 ± 8.4	34.9 ± 6.9	.76
AIx@HR75 (%)	10.6 ± 11.7	2.5 ± 11.1	.01	23.9 ± 12.4	18.6 ± 9.2	.03	28.3 ± 7.3	28.9 ± 5.7	.72
ED (m/s)	38.3 ± 5.1	36.0 ± 4.6	.06	35.4 ± 3.9	36.1 ± 3.7	.38	34.7 ± 3.7	34.9 ± 4.7	.87
SEVR (%)	141.6 ± 29.3	156.0 ± 33.1	.07	158.7 ± 26.2	155.6 ± 23.6	.53	158.1 ± 24.6	161.4 ± 31.0	.66
PWV (m/s)	5.4 ± 0.7	5.1 ± 0.8	.16	6.7 ± 1.1	6.5 ± 1.0	.32	8.0 ± 1.4	6.8 ± 1.3	.003
Operator index	94.6 ± 5.3	97.7 ± 2.8	.003	97.1 ± 3.5	95.2 ± 4.7	.03	96.5 ± 5.9	92.7 ± 6.6	.03

Abbreviations: AIx, augmentation index; AIx@HR75, augmentation index adjusted to a heart rate of 75 beats per minute; AP, augmentation pressure; BMI, body mass index; BP, blood pressure; bpm, beats per minute; BSA, body surface area; ED, ejection duration; MP, mean pressure; PP, pulse pressure; PWV, pulse wave velocity; SEVR, subendocardial viability ratio; T1, time at the first peak/shoulder during systole (outgoing pressure wave); T2, time at the second peak/shoulder during systole (reflected pressure wave); Tr, time to return of the reflected pressure; VVS, vasovagal syncope.

**FIGURE 2:**
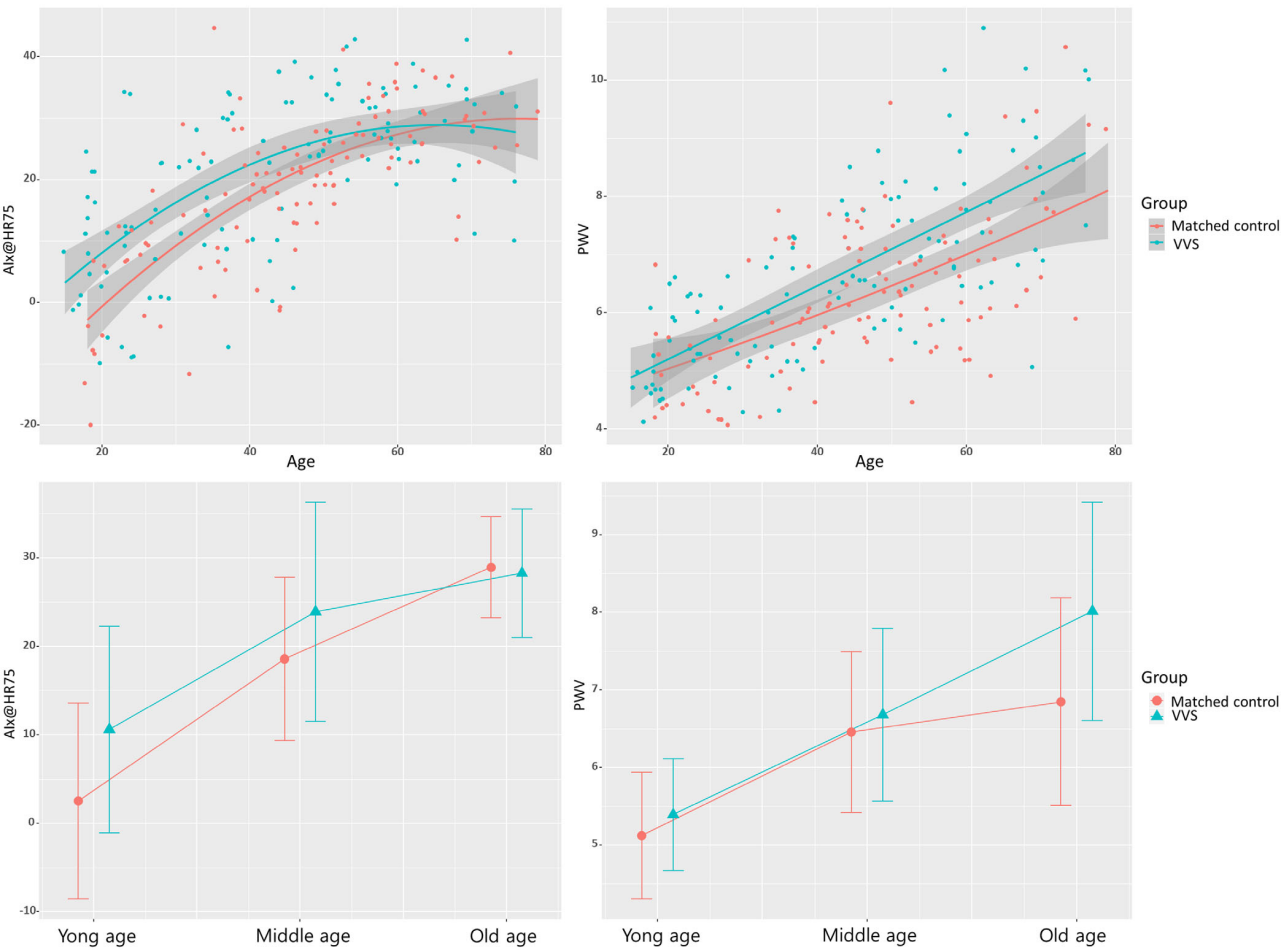
The trend of augmentation index adjusted to a heart rate of 75 beats/minute (AIx@HR75) and pulse wave velocity (PWV) according to the age distribution. AIx@HR75 greatly expanded as the age decreases in the vasovagal syncope (VVS) patients. By contrast, PWV was greater with increased age in the VVS patients

In the young age group, T1 was shorter in VVS patients (106.5 ± 12.6 m/s vs 118.0 ± 21.7 m/s, *P* = .02), whereas T2 and Tr were comparable. By contrast, in the middle age group, T2 was significantly longer in the VVS group (238.2 ± 17.8 m/s vs 229.1 ± 20.5 m/s, *P* = .03), whereas T1 and Tr showed no significant difference between the two groups. In the old age group, T1, T2, and Tr did not show significant differences between the two groups. AIx@HR75 was greater in VVS patients than in the control group in the young age group and middle age group (10.6 ± 11.7% vs 2.5 ± 11.1%, *P* = .01; 23.9 ± 12.4% vs 18.6 ± 9.2%, *P* = .03, respectively), whereas VVS patients and the control group were comparable in the old age group (*P* = .72). Contrary to AIx@HR75 results, PWV was greater in VVS patients in the old age group (8.0 ± 1.4 m/s vs 6.8 ± 1.3 m/s, *P* = .003) and showed no significant differences in the young and middle age groups. The value and trend of AIx@HR75 and PWV according to the age distribution are demonstrated in Figure 2.

### 3.4 VVS vs negative HUT test control subjects

We also compared PWA parameters between VVS patients and HUT negative control subjects (Table S2). Although there was no difference in age between the two groups, we performed matched analysis because gender, height, and weight were significantly different. In a matched analysis, T1, T2, and Tr were comparable between the two groups. However, AIx@HR75 was significantly higher in VVS patients (18.7 ± 13.1% vs 10.2 ± 13.8%, *P* = .01). In PWV, there was no significant difference (7.0 ± 1.6 m/s vs 6.5 ± 1.3 m/s, *P* = .14).

## 4 DISCUSSION

The purpose of this study was to investigate the difference in vascular properties between patients with VVS and healthy subjects using PWA. We observed significant changes in the aortic pressure waveform in patients with VVS. The VVS group had greater aortic stiffness than the control group. Our results indicated that VVS patients have different vascular properties compared to healthy individuals, which supports our hypothesis. Unlike previous studies, our study has novelty in that it showed a difference in vascular properties even after correcting for factors that could affect vascular waveform.^14^, ^15^, ^16^

The arterial pressure waveform is determined by the left ventricular stroke volume, the physical properties of the arterial wall, and blood pressure properties. A pressure waveform is initiated when blood exits the heart, and the pressure waveform proceeds faster than the blood flow. The pressure waveform progresses faster as the blood vessel becomes harder and smaller.^13^ Arterial stiffness refers to the degree of rigidity caused by the decrease in the elasticity of the arteries.^17^ The most important factor in determining arterial stiffness is age. With aging, changes in arterial wall tissues result in decreased elasticity and increased stiffness.^18^, ^19^ Arterial stiffness increases with an elevation of blood pressure and other diseases (eg, chronic heart failure, diabetes, and hyperlipidemia) as well as in conditions such as smoking and obesity.^15^, ^20^

PWA is a useful tool for noninvasive assessment of central hemodynamics and arterial elasticity indices that analyze the arterial pressure waveform.^8^ It is possible to determine important clinical parameters related to vascular stiffness, AIx, and PWV. PWV is the measurement of the speed of the pressure waves that travel along with the arterial segments, indicating the stiffness for a certain distance. On the other hand, AIx is defined as the change in the magnitude of PP caused by the reflected wave, a major measurement of hemodynamics associated with arterial stiffness. Because AIx is influenced by heart rate, the corrected index for heart rate 75 bpm (AI@HR75) is commonly used.^21^ Using PWV measurements, a clinician can gauge arterial stiffness that is reflective of the history of the patient's illness and can assess the effect of drug therapy in persons with normal ventricular ejection by measuring aortic AIx.^10^, ^12^, ^13^ These two parameters are known as independent predictors of cardiovascular disease.

In this study, we found that both PWV and AIx were higher in VVS than in the control group, indicating increasing vascular stiffness. It is important to emphasize that these modifications were observed at rest in a supine position, without any orthostatic stress. The shorter time needed to achieve the maximum systolic blood pressure may be a sign of increased vascular stiffness and impaired elasticity in the aorta. Nevertheless, subgroup analysis for age showed inconsistent results for AIx and PWV. In VVS patients, the difference in AIx value was significantly higher at young ages. However, the difference decreased with age, and there was no significant difference in the old age group. On the other hand, the PWV values did not differ between the two groups at a young age, whereas the increase in PWV was higher in the VVS patients with increasing age. These findings suggest the existence of different determinants of AIx and PWV. AIx can provide information on systemic arterial stiffness, PWV is derived from carotid β‐index, and it is an expression of local arterial stiffness.^22^ PWV was measured between carotid and femoral arterial sites with a Doppler flow velocity record technique; this again differs with respect to the site and method of recording pressure waveforms in the present study.^23^ We speculated that a decrease in systemic arterial elasticity would be a major factor of VVS at young ages, and impaired compensation of vascular tone due to increased peripheral arterial stiffness would be more dependent on VVS in elderly patients because arterial elasticity necessarily decreases with aging.

In a previous study, peripheral PWA detected a higher stiffness index and a longer time delay between the systolic blood pressure and diastolic blood pressure peak during finger arterial pressure monitoring.^24^ Another previously published report describes significant changes in the aortic elastic properties in patients with VVS measured using transthoracic echocardiography.^14^ The authors concluded that the aorta is an important modulator of cardiovascular homeostasis. Their results showed that aortic stiffness index and aortic elastic modulus were higher in patients with VVS compared to healthy individuals. Similarly, using the PWA in our study, we found increased aortic stiffness in VVS patients.

However, merely increased arterial stiffness cannot explain the entire mechanism of VVS because the current opinion suggests that the major pathophysiologic mechanism of VVS is autonomic dysfunction.^4^, ^5^, ^25^, ^26^ While controlling the vasomotor function by the arterial baroreflex plays a major role in rapid hemodynamic adjustments to the upright posture, autonomic failure dysregulates these processes.^26^ Nevertheless, several previous studies suggest that arterial stiffness can play a role in a key mechanism of syncope. One previous study demonstrated that patients with syncopal attacks showed increased arterial wave reflection compared to the control group, suggesting that greater arterial wave reflection implies higher carotid arterial and central aorta pressures that may cause a greater decline of baroreceptor function.^15^ Other studies have shown that impaired arterial elastic properties may interfere with the baroreceptor function and lead to diminished neuronal discharge of the vagal nerves or disability of the autonomic nervous system to activate the compensatory reflexes, resulting in impaired vascular elastic properties.^27^, ^28^ Therefore, as previous studies consistently suggest a significant correlation between impaired elastic properties and baroreceptor dysfunction,^15^, ^27^, ^28^ our study proposes that autonomic dysregulation results in impairment of arterial elastic properties in patients with VVS and leads to greater arterial stiffness.

To summarize, our data support the notion that impaired aortic function as increased aortic stiffness results in loss of compensation during hemodynamic alteration, impairing the circulation of blood through the cerebral vasculature. To date, there have been no data available to determine whether these modifications have a functional or structural nature.

Some limitations of our study should be noted. First, HUT was not performed on all healthy control subjects. Therefore, there is a possibility of selection bias because VVS was not completely excluded from healthy control subjects. Nevertheless, despite the small numbers, the results were consistent, even compared with the HUT negative healthy control subjects. This result can be thought to empower the validity of our suggestion. Second, PWA is not a tool for accessing sympathetic activity and peripheral vascular resistance. Therefore, it could not evaluate sympathetic activity or baroreceptor sensitivity and was limited to access an exact hemodynamic property. Third, although AIx well reflects systemic vascular resistance, it easily affected by blood pressure, heart rate, sex, age, height, and drugs that affect blood vessels. Therefore, it is not enough to determine what the pathophysiologic consequence of the study results could be. However, for this reason, we used propensity score matching analysis, and we found that the AIx is consistently increased in VVS patients. Thus, it is further in support of our assertion for pathophysiological relevance. Forth, because of the relatively small number of patients and the single referral tertiary institute data, our study participants may be a skewed and selected population, rather than representing the general population. For this reason, we instituted strict inclusion and exclusion criteria. Our findings would be validated in a larger cohort with multi‐center studies.

In conclusion, our study found that patients with VVS have altered aortic pressure waveforms with greater arterial stiffness compared to healthy controls. These findings will help determine the mechanisms involved in the pathophysiology of VVS. Further research is needed to provide more robust information about the direct mechanistic relationship between arterial stiffness and autonomic function in VVS.

## CONFLICT OF INTEREST

The authors have no conflict of interest to declare.

## Supporting information


**Appendix** **S1:** Supporting information
