# Supplementary material for: An analysis of vascular properties using pulse wave analysis in patients with vasovagal syncope
Source: Clin Cardiol. 2020 Jun 18;43(7):781–8. doi: 10.1002/clc.23380 (PMC7368349; doi:10.1002/clc.23380)
Supplement: Supplementary file 1 — Appendix S1: Supporting information [file CLC-43-781-s001.docx]

**Supplementary Materials**

**An analysis of vascular properties using pulse wave analysis in patients with vasovagal syncope**

**Running Head:** Pulse wave analysis of vascular properties

Ji-Hun Jang, Jin-Hee Park, Kyu-Yong Ko, Yong-Soo Beak, Sung-Woo Kwan, Sang-Don Park, Sung-Hee Shin, Seong-Ill Woo, Jun Kwan, Dae-Hyeok Kim

1. Supplementary Table S1

2. Supplementary Table S2

| Supplementary Table S1. Baseline characteristics and pulse wave analysis according to VVS type. | | | | |
| --- | --- | --- | --- | --- |
| Parameters | VVS type | | | P value |
|  | Type I, mixed  (n = 14) | Type II, cardio-inhibitory  (n = 12) | Type III, vasodepressor  (n = 85) |  |
| **Baseline characteristics** |  |  |  |  |
| Age (years) | 40 ± 15 | 45 ± 17 | 43 ± 18 | 0.79 |
| Female, n (%) | 10 (71.4%) | 8 (66.7%) | 54 (64.3%) | 0.94 |
| Height (cm) | 165.4 ± 8.6 | 163.9 ± 9.5 | 163.3 ± 9.2 | 0.43 |
| Weight (kg) | 60.6 ± 10.0 | 65.0 ± 9.6 | 60.0 ± 9.5 | 0.47 |
| BSA (m²) | 1.7 ± 0.2 | 1.7 ± 0.2 | 1.6 ± 0.2 | 0.43 |
| BMI (kg/m²) | 22.1 ± 2.6 | 24.2 ± 2.5 | 22.4 ± 2.7 | 0.84 |
| Smoking (%) | 2 (14.3%) | 0 (0.0%) | 8 (9.5%) | 0.45 |
| Heart rate, bpm | 71.2 ± 14.7 | 61.2 ± 9.8 | 65.4 ± 9.7 | 0.16 |
| **Pulse wave analysis** |  |  |  |  |
| Radial BP (mmHg) |  |  |  |  |
| Systolic | 118.1 ± 12.7 | 122.2 ± 13.7 | 117.7 ± 14.6 | 0.49 |
| Diastolic | 72.4 ± 9.4 | 75.5 ± 7.1 | 71.7 ± 8.5 | 0.47 |
| MP | 89.1 ± 11.6 | 93.2 ± 8.9 | 88.6 ± 10.2 | 0.30 |
| PP | 45.7 ± 7.4 | 46.7 ± 12.7 | 45.9 ± 11.2 | 0.97 |
| Aortic BP (mmHg) |  |  |  |  |
| Systolic | 107.6 ± 14.6 | 114.3 ± 15.9 | 108.1 ± 15.2 | 0.35 |
| Diastolic | 73.6 ± 9.3 | 76.4 ± 7.1 | 72.8 ± 8.5 | 0.46 |
| MP | 89.1 ± 11.6 | 93.2 ± 8.9 | 88.6 ± 10.2 | 0.30 |
| PP | 34.1 ± 8.7 | 37.9 ± 14.4 | 35.3 ± 11.3 | 0.97 |
| T1 (m/s) | 108.1 ± 11.5 | 108.9 ± 12.0 | 106.5 ± 12.1 | 0.69 |
| T2 (m/s) | 218.7 ± 35.1 | 236.9 ± 34.2 | 230.6 ± 24.3 | 0.45 |
| Tr (m/s) | 142.8 ± 13.7 | 142.8 ± 15.7 | 143.6 ± 15.4 | 0.93 |
| P1 height (mmHg) | 24.9 ± 4.1 | 26.2 ± 6.3 | 25.7 ± 6.1 | 0.94 |
| AP | 9.1 ± 8.1 | 11.8 ± 9.5 | 9.5 ± 7.3 | 0.62 |
| Aortic AIx (%) | 23.2 ± 18.3 | 27.2 ± 16.2 | 24.5 ± 13.2 | 0.74 |
| AIx@HR75 (%) | 21.4 ± 15.2 | 20.5 ± 14.9 | 20.2 ± 12.6 | 0.94 |
| ED (m/s) | 38.2 ± 6.4 | 34.8 ± 4.3 | 36.1 ± 4.2 | 0.34 |
| SEVR (%) | 143.5 ± 34.6 | 161.6 ± 25.3 | 152.9 ± 27.0 | 0.47 |
| PWV (m/s) | 6.2 ± 1.1 | 7.2 ± 1.6 | 6.6 ± 1.6 | 0.34 |
| Operator index | 93.9 ± 5.7 | 97.7 ± 2.3 | 96.2 ± 5.1 | 0.15 |
| AIx, augmentation index; AIx@HR75, augmentation index adjusted to a heart rate of 75 beats per minute; AP, augmentation pressure; BMI, body mass index; BP, blood pressure; bpm, beats per minute; BSA, body surface area; ED, ejection duration; MP, mean pressure; PP, pulse pressure; PWV, pulse wave velocity; SEVR, subendocardial viability ratio; T1, time at the first peak/shoulder during systole (outgoing pressure wave); T2, time at the second peak/shoulder during systole (reflected pressure wave); Tr, time to return of the reflected pressure; VVS, vasovagal syncope | | | | |

| Supplementary Table S2. Baseline characteristics and pulse wave analysis by VVS group and healthy control subjects with HUT negative in the overall study population and 1:1 matched study population. | | | | | | | |
| --- | --- | --- | --- | --- | --- | --- | --- |
| Parameters | Overall | | |  | Matched | | |
|  | VVS  (n = 111) | HUT test (-)  (n = 39) | P value |  | VVS  (n=39) | HUT test (-)  (n = 39) | P value |
| **Baseline characteristics** |  |  |  |  |  |  |  |
| Age (years) | 43 ± 18 | 43 ± 17 | 0.79 |  | 44 ± 17 | 43 ± 17 | 0.97 |
| Female, n (%) | 72 (64.9%) | 13 (33.3%) | 0.001 |  | 14 (35.9%) | 13 (33.3%) | 0.99 |
| Height (cm) | 163.7 ± 9.1 | 168.4 ± 9.5 | 0.01 |  | 167.1 ± 8.2 | 168.4 ± 9.5 | 0.54 |
| Weight (kg) | 60.7 ± 9.6 | 68.0 ± 13.1 | 0.001 |  | 66.5 ± 10.2 | 68.0 ± 13.1 | 0.58 |
| BSA (m²) | 1.7 ± 0.2 | 1.8 ± 0.2 | <0.001 |  | 1.8 ± 0.2 | 1.8 ± 0.2 | 0.57 |
| BMI (kg/m²) | 22.6 ± 2.7 | 23.8 ± 3.2 | 0.03 |  | 23.7 ± 2.6 | 23.8 ± 3.2 | 0.95 |
| Smoking (%) | 10 ( 9.0%) | 5 (12.8%) | 0.71 |  | 5 (12.8%) | 5 (12.8%) | 0.99 |
| Heart rate, bpm | 65.8 ± 10.6 | 65.6 ± 10.8 | 0.99 |  | 63.7 ± 11.2 | 65.6 ± 10.8 | 0.45 |
| **Pulse wave analysis** |  |  |  |  |  |  |  |
| Radial BP (mmHg) |  |  |  |  |  |  |  |
| Systolic | 118.3 ± 14.2 | 119.9 ± 13.5 | 0.40 |  | 118.8 ± 9.5 | 119.9 ± 13.5 | 0.69 |
| Diastolic | 72.3 ± 8.5 | 73.8 ± 8.1 | 0.33 |  | 72.3 ± 8.0 | 73.8 ± 8.1 | 0.40 |
| MP | 89.3 ± 10.3 | 89.5 ± 9.5 | 0.75 |  | 89.2 ± 8.6 | 89.5 ± 9.5 | 0.89 |
| PP | 46.0 ± 10.8 | 46.0 ± 10.5 | 0.89 |  | 46.4 ± 8.3 | 46.0 ± 10.5 | 0.51 |
| Aortic BP (mmHg) |  |  |  |  |  |  |  |
| Systolic | 108.8 ± 15.2 | 107.5 ± 12.8 | 0.96 |  | 108.9 ± 11.5 | 107.5 ± 12.8 | 0.61 |
| Diastolic | 73.4 ± 8.5 | 74.8 ± 8.4 | 0.37 |  | 73.4 ± 8.1 | 74.8 ± 8.4 | 0.44 |
| MP | 89.3 ± 10.3 | 89.5 ± 9.5 | 0.75 |  | 89.2 ± 8.6 | 89.5 ± 9.5 | 0.89 |
| PP | 35.5 ± 11.3 | 32.7 ± 9.1 | 0.29 |  | 35.5 ± 9.0 | 32.7 ± 9.1 | 0.20 |
| T1 (m/s) | 106.9 ± 12.0 | 116.7 ± 13.7 | <0.001 |  | 111.9 ± 13.1 | 116.7 ± 13.7 | 0.10 |
| T2 (m/s) | 229.8 ± 27.0 | 221.9 ± 26.6 | 0.06 |  | 231.5 ± 30.8 | 221.9 ± 26.6 | 0.14 |
| Tr (m/s) | 143.3 ± 15.1 | 152.3 ± 14.4 | 0.001 |  | 147.8 ± 15.0 | 152.3 ± 14.4 | 0.19 |
| P1 height (mmHg) | 25.6 ± 5.9 | 26.7 ± 6.4 | 0.46 |  | 26.3 ± 4.6 | 26.7 ± 6.4 | 0.89 |
| AP | 9.8 ± 7.6 | 5.7 ± 6.4 | 0.003 |  | 9.2 ± 7.0 | 5.7 ± 6.4 | 0.02 |
| Aortic AIx (%) | 24.7 ± 14.2 | 15.3 ± 15.6 | 0.001 |  | 23.7 ± 13.9 | 15.3 ± 15.6 | 0.02 |
| AIx@HR75 (%) | 20.5 ± 13.1 | 10.2 ± 13.8 | <0.001 |  | 18.7 ± 13.1 | 10.2 ± 13.8 | 0.01 |
| ED (m/s) | 36.2 ± 4.6 | 35.8 ± 4.5 | 0.69 |  | 35.3 ± 4.9 | 35.8 ± 4.5 | 0.68 |
| SEVR (%) | 152.5 ± 27.8 | 157.4 ± 31.6 | 0.36 |  | 159.6 ± 30.9 | 157.4 ± 31.6 | 0.77 |
| PWV (m/s) | 6.6 ± 1.5 | 6.5 ± 1.3 | 0.76 |  | 7.0 ± 1.6 | 6.5 ± 1.3 | 0.14 |
| Operator index | 96.0 ± 5.0 | 97.5 ± 3.1 | 0.23 |  | 97.3 ± 3.8 | 97.5 ± 3.1 | 0.86 |
| AIx, augmentation index; AIx@HR75, adjusted to a heart rate of 75 beats per minute; AP, aortic augmentation; BMI, body mass index; BP, blood pressure; bpm, beats per minute; BSA, body surface area; ED, ejection duration; HUT, head-up tilt; MP, mean pressure; PP, pulse pressure; PWV, pulse wave velocity; SEVR, subendocardial viability ratio; T1, time at the first peak/shoulder during systole (outgoing pressure wave); T2, time at the second peak/shoulder during systole (reflected pressure wave); Tr, time to return of the reflected pressure; VVS, vasovagal syncope | | | | | | | |
